# Is Croupous Pneumonia a Specific Infectious Disease?

**Published:** 1879-09-20

**Authors:** S. H. Anderson

**Affiliations:** Mo.


### *IS CROUPOUS PNEUMONIA A SPECIFIC INFECTIOUS DISEASE?*

BY S. H. ANDERSON, M.D., OF MO.

In June number of the Record appeared a very interesting article on croupous form of pneumonia—interesting from what I must regard as *novel* doctrine as to pathological nature of this *very* common affection.

Dr. Hobbs assumes that croupous pneumonia is specific and, therefore, infectious in character, than which in my humble opinion there can be no more dangerous and mischievous teaching. For if the disease under consideration be a specific infectious disease, the treatment must necessarily be reduced to a routine, expectant and non-effective plan.

No physician, for instance, well up in his profession would dare treat a case of typhoid fever as vigorously as he would an open, sthenic inflammation of the lungs. On the contrary, would he not lay aside his veratrum, aconite, antimony, etc., and resort to so-called specifics, as quinine, wine, generous diet, etc.?

But *is* croupous pneumonia a specific infectious disease? On the contrary, I believe it to be, for the best of reasons, a sthenic inflammation of the lungs, in a vast majority of cases the best type of the inflammatory process known to pathologists.

Shortly expressed, what are the pathological conditions necessarily present to determine an attack of acute croupous pneumonia? I will here remark that it is an error to suppose, as is often done, that the most common subjects of pneumonitis are the robust and well-fed. On the contrary, it will usually be found that those persons more subject to an attack of this disease are the ill-nourished, those more exposed to the vicissitudes of the weather and badly clothed; in a word those persons whose systems are loaded down with the effete materials of tissue-metamorphosis; whose kidneys, bowels, skin, etc., have ceased to properly eliminate from the body this poisonous principle which, on the application of an exciting cause, as cold and wet, results in an effort of nature to rid the system of this offending cause. What offending cause? I answer, an unnatural proportion of white blood-cells or corpuscles; blood-cells which, from innervation, etc., have not been carried up to physiological blood-cells; cells which have, as it were, *aborted*, and are, therefore, unfit for the purposes of nutrition; cells which are in fact *dead* material, and, as such, must be eliminated from the body before health can be re-established. And what is the fever of pneumonia but an effort to *vitalize* those aborted cells, in order to their elimination?

If this be so, it is idle to talk about infection. And, in regard to infectious diseases, they never arise *de novo*. This may sound dogmatic, but no one can prove the contrary. There are many ways unknown to us by which infection may be conveyed, the infection of typhoid fever, for instance. Many local epidemics of this disease have been shown to have been carried by the drinking of water from rivers and creeks, miles below, where the dejections of typhoid patients have been emptied into such stream. And again by drinking of milk brought from houses where cases of the disease were known to exist. Hence, no man can prove that all cases of known infectious diseases originate in any other way than by the reception of the specific poison into the system of the victim of such disease.

And here I will remark that I believe croupous and catarrhal pneumonia to be merely exaggerated forms of each other.

Another strong proof of the non-infectious nature of the disease under consideration, is the indubitable fact that it can be and has been again and again aborted by early and effective treatment. In this statement I am amply sustained by a host of witnesses; not mere closet theorists, but by men who have grown gray in the arduous duties of active professional life. And I can truly say, after the experiences culminating in a practice of a quarter of a century, no facts in this eventful period have given me more reasons for self-gratulation than the assurances I have been able to give my patients laboring under this dangerous affection, of speedy relief, arising from the knowledge I had of being able to abort the attack, to strangle it, in its incipient stage. That I have done so many, many times, I cannot allow myself to doubt. Therefore, I arrive at the conclusion that to teach that this disease—a purely inflammatory disease—is infectious, must be a dangerous doctrine, at best to the young members of the profession.

In regard to treatment, I know of no substitute for veratrum viride. It is the grand, great eliminator *par excellence*. Of course, there are many auxiliary remedies, but I, for one, place my main reliance on veratrum viride. Perhaps this is my hobby; be it so! But a friend in need is a friend indeed! As in conjunction with other remedies, I have found veratrum viride to fulfill indications in the treatment of many and various diseases essentially differing in nature from pneumonia, I, therefore, conclude that its powers are, as yet, but little known.

